# Genetic association and stress mediated down-regulation in trabecular meshwork implicates *MPP7* as a novel candidate gene in primary open angle glaucoma

**DOI:** 10.1186/s12920-016-0177-6

**Published:** 2016-03-22

**Authors:** Mansi Vishal, Anchal Sharma, Lalit Kaurani, Giovanna Alfano, Suddhasil Mookherjee, Kiran Narta, Jyoti Agrawal, Iman Bhattacharya, Susanta Roychoudhury, Jharna Ray, Naushin H. Waseem, Shomi S. Bhattacharya, Analabha Basu, Abhijit Sen, Kunal Ray, Arijit Mukhopadhyay

**Affiliations:** Molecular and Human Genetics Division, CSIR-Indian Institute of Chemical Biology, Kolkata, 700032 India; Genomics & Molecular Medicine, CSIR-Institute of Genomics & Integrative Biology, Mathura Road (near Sukhdev Vihar), New Delhi, 110025 India; Academy of Scientific and Innovative Research (AcSIR), New Delhi, 110025 India; UCL Institute of Ophthalmology, London, EC1V 9EL UK; Premas Biotech Pvt. Ltd, Gurgaon, Haryana 122050 India; Cancer Biology and Inflammatory disorder division, CSIR-Indian Institute of Chemical Biology, Kolkata, 700032 India; S. N. Pradhan Centre for Neurosciences, University of Calcutta, Kolkata, 700019 India; National Institute of Biomedical Genomics, Kalyani, 741251 India; Dristi Pradip, Kolkata, 700068 India

**Keywords:** POAG, IOP, MPP7, GWA, HTM, LD, GEO, SNP

## Abstract

**Background:**

Glaucoma is the largest cause of irreversible blindness affecting more than 60 million people globally. The disease is defined as a gradual loss of peripheral vision due to death of Retinal Ganglion Cells (RGC). The RGC death is largely influenced by the rate of aqueous humor production by ciliary processes and its passage through the trabecular meshwork (TM) in the anterior part of the eye. Primary open angle glaucoma (POAG), the most common subtype, is a genetically complex disease. Multiple genes and many loci have been reported to be involved in POAG but taken together they explain less than 10 % of the patients from a genetic perspective warranting more studies in different world populations. The purpose of this study was to perform genome-wide search for common variants associated with POAG in an east-Indian population.

**Methods:**

The study recruited 746 POAG cases and 697 controls distributed into discovery and validation cohorts. In the discovery phase, genome-wide genotype data was generated on Illumina Infinium 660 W-Quad platform and the significant SNPs were genotyped using Illumina GGGT assay in the second phase. Logistic regression was used to test association in the discovery phase to adjust for population sub-structure and chi-square test was used for association analysis in validation phase. Publicly available expression dataset for trabecular meshwork was used to check for expression of the candidate gene under cyclic mechanical stress. Western blot and immunofluorescence experiments were performed in human TM cells and murine eye, respectively to check for expression of the candidate gene.

**Results:**

Meta-analysis of discovery and validation phase data revealed the association of rs7916852 in MPP7 gene (p = 5.7x10^−7^) with POAG. We have shown abundant expression of MPP7 in the HTM cells. Expression analysis shows that upon cyclic mechanical stress *MPP7* was significantly down-regulated in HTM (Fold change: 2.6; p = 0.018). MPP7 protein expression was also found to be enriched in the ciliary processes of the murine eye.

**Conclusion:**

Using a genome-wide approach we have identified *MPP7* as a novel candidate gene for POAG with evidence of its expression in relevant ocular tissues and dysregulation under mechanical stress possibly mimicking the disease scenario.

**Electronic supplementary material:**

The online version of this article (doi:10.1186/s12920-016-0177-6) contains supplementary material, which is available to authorized users.

## Background

Glaucoma is the second largest cause of blindness after cataract [1] and it is the leading cause of irreversible blindness worldwide. Primary Open Angle Glaucoma (POAG), a multifactorial complex disease, is the most common subtype. The disease is characterized by progressive loss of peripheral vision due to death of retinal ganglion cells and a characteristic abnormal appearance of optic nerve head [2]. Ocular risk factors for this disease are high Intra-Ocular Pressure (IOP), thinner Central Corneal Thickness (CCT) and myopia [3]. High IOP (>21 mm of Hg) is the most important risk factor of POAG, although it is neither necessary nor sufficient for the disease onset [4]. However, the most effective treatment strategy till date is IOP management and it has proven to be beneficial even for normal tension glaucoma patients (IOP < 21 mm Hg) [5, 6].

The balance between production of aqueous humor by ciliary body and outflow through the trabecular meshwork determines IOP [4]. It has been shown that highly penetrant genetic mutations in *MYOC* gene can result in reduced filtration rates of aqueous humor due to protein aggregation and sequestration due to misfolding causing elevation of IOP [7, 8].

The genetic etiology of POAG is poorly understood. Family based linkage analyses have revealed 17 linked loci for POAG of which six genes have been identified (OMIM 137760). Candidate gene studies have suggested multiple susceptibility loci to be associated with this disease [3]. A total of 11 Genome Wide Association Studies (GWAS) have been reported for POAG to date from different populations of the world. About 14 GWA studies on optic disc parameters are reported, namely Intra-Ocular Pressure (IOP), Vertical Cup-Disc Ratio (VCDR), Central Corneal Thickness (CCT) and Optic disc area [3]. From these studies, a few loci were replicated in populations of different ancestries [3, 9, 10]. Among these, studies in Indian population do not show evidence of association for CDKN2B-AS1 [11] and PLXDC2 loci [12] probably indicating a different genetic structure of this population. There is no data for other loci and no unbiased genetic screen has been performed for POAG from this part of the world. Here, we report a genome-wide search for common variants associated with POAG in a large population residing in the West Bengal state of India.

## Methods

### Selection of study subjects and sample preparation

A total of 364 POAG cases and 365 controls were selected for the discovery phase of the study and 382 cases and 332 controls were selected for the replication cohort. The patients were diagnosed through clinical ocular and systemic examinations. The inclusion and exclusion criteria for samples were the same as reported earlier [13]. Briefly, the patients were recruited if they were positive for 2 out of the 3 criteria, namely, Intra-ocular pressure (IOP) >21 mm of Hg, glaucomatous field damage and significant cupping of the optic disc. Individuals with ocular hypertension and with any history of inflammation or ocular trauma (past & present) were excluded from this study.

Controls were selected without any history of ocular disease and wherever possible were tested negative for POAG by means of routine eye examination for glaucoma as described above. The study protocol adhered to the tenets of the Declaration of Helsinki and was approved by the Institutional Review Board.

Peripheral blood was collected with EDTA from the POAG patients and controls. A written informed consent was obtained from each individual. Genomic DNA was prepared from fresh whole blood using the PAXgene blood DNA isolation kit (Qiagen, Hilden, Germany) according to the manufacturer’s protocol. The DNA was dissolved in TE (10 mM Tris–HCl, 1 mM EDTA, pH 8.0).

### Genome-wide genotyping and quality control for discovery phase

In the discovery phase, genome-wide genotyping was done using Illumina Human660W-Quad chip (Illumina Inc., San Diego, CA, USA) following the manufacturer’s protocol. Genotype data of SNPs were obtained from Genome Studio version 2011.1. Gentrain score >0.3 was taken as threshold for cluster quality of SNPs. Duplicate samples and close relatives (first degree relatives) were removed by Identity-by-state analysis in PLINK (version 1.06). Samples with call rate >98 % and SNPs with call rate >95 % were retained. Subsequently, SNPs with minor allele frequency <0.01 in controls and SNPs which do not follow Hardy-Weinberg equilibrium (HWE p < 0.01) were removed. The genomic inflation factor (λ) in the discovery cohort was 1.06 suggesting population sub-structure. Three outlier samples were removed from the final analysis after multidimensional scaling (see Additional file 1) and p-values were adjusted for remaining stratification using values of four components as covariates by logistic regression. The inflation factor for the adjusted p-values was observed to be 1.01. We have also performed chi-square based statistics for which the data is provided in Additional file 2.

### Linkage disequilibrium (LD)-based SNP clumping

To assess the confidence of association of independent loci, we performed genome-wide LD-based clumping. The criteria for clumping was based on index SNP (p < 0.0001), clumped SNPs (p < 0.01), linkage disequilibrium (r^2^ > 0.5) and physical genomic distance from the index SNP of 250 Kb [14].

### Genotype imputation

Further to increase the genomic coverage for the regions we imputed SNP data using MACH (version 1.0.16). The reference populations for imputation were the combined HAPMAP phase 3 data of CEU (Utah residents with European ancestry) and GIH (Gujarati Indians in Texas, Houston) [15]. The representative genotype and allele error rates are given in Additional file 3.

### Targeted genotyping and quality control for validation phase

The SNPs with p < 10^−3^ and the associated clumped SNPs from 31 clumps after imputation were taken forward for validation in an independent cohort. Thus, 514 SNPs were genotyped using the Illumina GoldenGate genotyping assay (Illumina GGGT assay) in 382 cases and 332 controls from the same population background. As mentioned above for the discovery phase, here also we have performed QC checks for call rate, MAF and HWE. Additionally, we removed six SNPs which showed significant allele frequency difference (Bonferroni-adjusted p-value < 0.05) between controls of discovery and validation cohorts (see Additional file 4). A total of 494 SNPs passed all quality checks (see Methods section) and were tested for association using chi-squared test in 319 cases and 297 controls. A total of 37 samples were genotyped in duplicate to check the accuracy which showed a concordance of >98.8 % (see Additional file 5).

### Statistical analysis

Statistical analysis for quality control, chi-square test of association, logistic regression for adjustment of p-values and multi-dimensional scaling for population stratification were performed using PLINK version 1.07 [14]. LD-based clumping was done using PLINK (version 1.06). Meta-analysis of discovery and replication phases was done using METAL [16]. Manhattan plots were created in qqman package of ‘R’ [17] and regional association plot was created using LOCUSZOOM [18].

### Analysis of GEO expression dataset

Microarray expression data of human trabecular meshwork (HTM) cell cultures was taken from publicly available gene expression omnibus (GEO) dataset (*GSE14768*). HTM cell cultures were obtained from cadaver eyes of three donors, 48 h post-mortem, with no history of eye diseases. The cells were subjected to cyclic mechanical stress and non-stressed parallel control cultures were incubated under the same conditions in the absence of stress. Data were analyzed using GEO2R online tool to check for the differential gene expression and tested for significance using *T*-test.

### Western blotting of *MPP7* in HTM cells

HTM cells were grown in six well plates. Cell lysates were prepared in RIPA lysis and extraction buffer (Life technologies, Carlsbad, CA, USA). Total protein estimation was performed using Qubit protein assay kit (Life technologies, Carlsbad, CA, USA). Thirty microgram of protein was electrophoresed in 10 % SDS-polyacrylamide gel (MiniPROTEAN III; BioRad, Herucles, CA) and transferred onto a PVDF membrane (Hybond-P; GE Healthcare, Bedford, UK) by electroblotting using wet transfer unit (BioRad laboratories, Hercules, CA). Membranes were then blocked in 5 % BSA in TBST [25 mM Tris–HCl (pH 7.5), 150 mM NaCl, 0.05 % tween-20] for one hour at room temperature and incubated with respective primary antibody [Rabbit anti-MPP7 polyclonal antibody (1:1000) (Abcam, UK) raised against synthetic peptide sequence 340- and Anti-β actin (1:2000) antibody (Sigma-Aldrich, USA) as control] overnight at 4 °C. The membranes were washed thrice with TBST [25 mM Tris–HCl (pH 7.5), 150 mM NaCl, 0.05 % Tween 20] for 10 mins interval followed by incubation with secondary antibody conjugated with HRP [anti-rabbit (1:2000)] (Bangalore Genei, India) for 1 h at room temperature. The secondary antibody was washed thrice with TBST. The blot was developed by the chromogenic substrate 3,3’-Diaminobenzidine (Sigma-Aldrich, USA).

### MPP7 expression pattern in mouse eye by Immunofluorescence

Expression studies were performed on P15 (2 weeks after birth) mouse eye. Eyes were obtained from C57/BL6 wild-type animals, fixed in 4 % paraformaldehyde, cryoprotected with a sucrose gradient (10-30 %) and embedded in optimal cutting temperature (OCT) compound (VWR, UK). Cryosections (10 μm) were treated at high temperature in 0.01 M Citrate buffer for antigen retrieval. Sections were blocked (2 h at room temperature) and hybridized (18 h at 4 °C) with PBS containing 5 % donkey serum (Sigma-Aldrich, MO, USA), 6 % bovine serum albumin (BSA) and 0.3 % Tween 20. A goat MPP7 antibody (N-15, sc-163089, Santa Cruz Biotechnology, Inc., Texas, USA) diluted to 1:50 and a secondary donkey anti-goat antibody conjugated with AlexaFluor488 (Molecular Probe, Invitrogen) diluted to 1:500 were used. Nuclei were stained (10 min at room temperature) with 4', 6-Diamidino-2-Phenylindole Dihydrochloride (DAPI, 1:5000). Slides were viewed on a Carl Zeiss S100 inverted microscope.

## Results

In this study we recruited a total of 1443 samples from a large population residing in the state of West Bengal in eastern part of India. The average age of patients was 54.32 ± 14.62 years and 51.61 ± 11.40 years for controls. The average IOP for the patients was 21.99 ± 7.88 mm of Hg.

### Genome-wide association study reveals association of novel loci for POAG

**Fig. 1:**
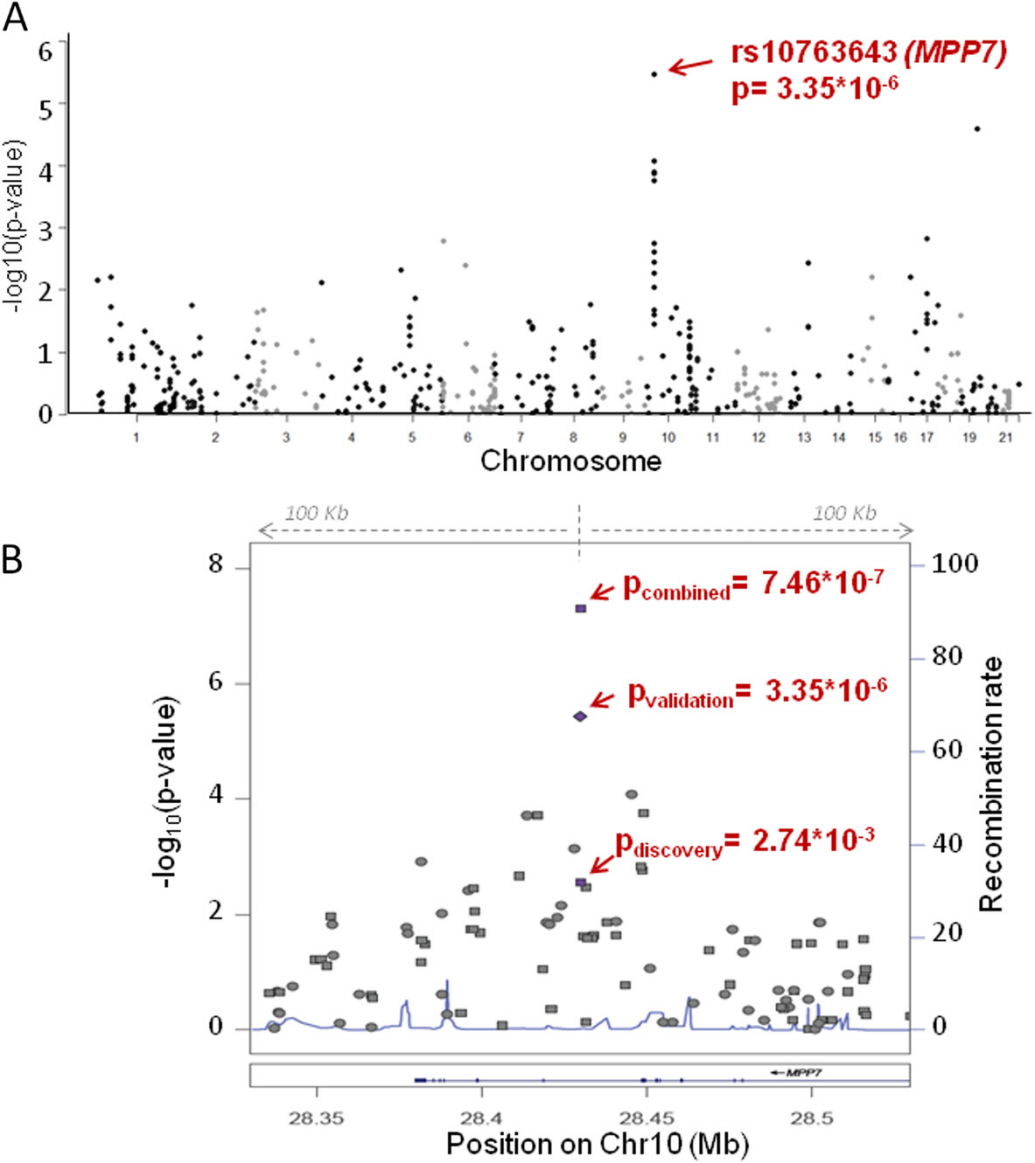
Association results of discovery and validation cohort. **a** Manhattan plot representing chi-square p-values for 494 genotyped SNPs in the validation phase (319 POAG cases and 297 controls). X-axis represents different chromosomes and Y-axis represents –log_10_ transformed p-values. The MPP7 SNP (rs10763643) having the lowest association in the validation phase is indicated. **b** Regional association plot for rs10763643 of MPP7 gene with 100 Kb upstream and downstream regions. The data of genotyped SNPs are denoted as circles while the imputed data is denoted as squares. The arrows represent association of rs10763643 in discovery, validation and the meta analysis. The left Y-axis represents –log_10_ p-values and the right Y-axis represents the recombination rate. X-axis represents position of SNPs on chromosome 10 (human genome build 36)

**Fig. 2:**
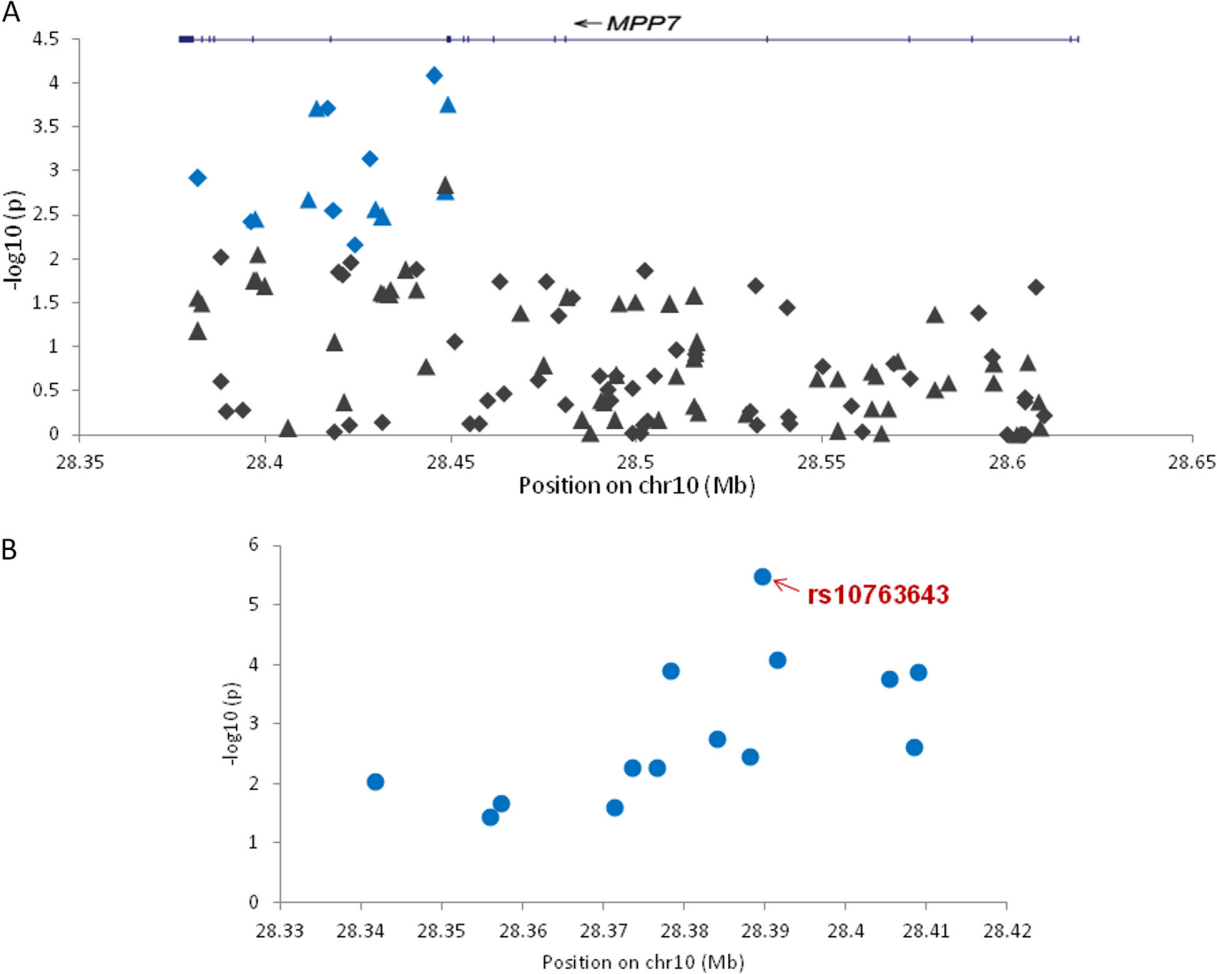
Association of *MPP7* SNPs in Discovery and validation phase. **a** The plot shows a total of 130 SNPs from *MPP7* locus in discovery phase. The genotyped SNPs are represented as diamonds and imputed SNPs are represented as triangles. The blue colour denotes 14 SNPs of MPP7 clump which were selected for validation phase. **b** This plot represents data of 14 MPP7 clump SNPs in the validation phase. All 14 SNPs have a p < 0.05. X-axis denotes the genomic position of SNPs in Mb (human genome build 36) and Y-axis represents –log_10_ p-values. The most significant SNP in the validation phase is marked with an arrow

The allelic association was tested on 347 cases and 354 controls for 521,873 autosomal SNPs (see Additional files 2 and 6) in the discovery phase and in the validation phase 494 SNPs were tested for association in another 319 cases and 297 controls from the same population (Fig. 1a, see methods). Meta-analysis of the entire data revealed most significant association of rs7916852 (p = 5.7x10^−7^, OR = 1.70) from MPP7. We observed association of 13 additional SNPs of MPP7 gene in the validation phase (meta-analysis p values ranging between 10^−7^ to 10^−3^; Table 1, see Additional file 2). Two more SNPs, rs10763644 and rs10763643, also showed the same magnitude of significance (Table 1). Interestingly, all the 14 SNPs were associated in the discovery phase as part of a single clump) (Fig. 2). It is worth highlighting that rs10763643, found to be one of the most associated SNPs in our genetic screen was originally obtained through imputation of genotypes from the HAPMAP and was experimentally validated in our cohort (Fig. 1, Table 1).

**Table 1:** Details of MPP7 SNPs associated with POAG in Indian population

SNP ID	Minor allele	Discovery cohort (347 cases/354 controls)			Validation cohort (319 cases/297 controls)			Meta-analysis (666 cases/651 controls)
		MAF (Cases/Controls)	p_logistic_	OR (95 % CI)	MAF (Cases/Controls)	P	OR (95 % CI)	P_meta_
rs7916852	C	0.54/0.43	9.19x10^−4^	1.54 (1.25-1.9)	0.61/0.5	1.71x10^−4^	1.54 (1.23-1.93)	5.7x10^−7^
rs10763644	G	0.54/0.44	1.24x10^−3^	1.53 (1.23-1.89)	0.61/0.5	1.33x10^−3^	1.55 (1.24-1.95)	6.18x10^−7^
rs10763643	A	0.48/0.4	0.018	1.4 (1.13-1.72)	0.58/0.44	3.35x10^−6^	1.7 (1.36-2.14)	7.46x10^−7^
rs4749305	A	0.48/0.4	0.020	1.39 (1.12-1.71)	0.55/0.44	8.42x10^−5^	1.57 (1.25-1.96)	9.92x10^−6^
rs11006851	G	0.48/0.4	0.023	1.39 (1.13-1.72)	0.55/0.44	1.26x10^−4^	1.55 (1.24-1.94)	1.57x10^−5^
rs10763642	A	0.4/0.49	1.9x10^−3^	0.69 (0.56-0.85)	0.34/0.42	3.51x10^−3^	0.71 (0.56-0.89)	2.03x10^−5^
rs10047289	A	0.4/0.49	3.9x10^−3^	0.71 (0.57-0.87)	0.34/0.43	2.44x10^−3^	0.7 (0.56-0.88)	2.92x10^−5^
rs7474568	A	0.46/0.36	1.82x10^−3^	1.52 (1.23-1.88)	0.5/0.43	5.42x10^−3^	1.38 (1.1-1.72)	3.03x10^−5^
rs4749301	A	0.46/0.36	1.8x10^−3^	1.52 (1.23-1.88)	0.5/0.42	5.42x10^−3^	1.38 (1.1-1.72)	3.03x10^−5^
rs3802520	A	0.36/0.45	6.7x10^−3^	0.7 (0.56-0.87)	0.33/0.4	9x10^−3^	0.73 (0.58-0.93)	1.68x10^−4^
rs7086023	A	0.4/0.33	0.040	1.36 (1.09-1.69)	0.45/0.36	1.78x10^−3^	1.44 (1.15-1.81)	2.58x10^−4^
rs12253376	A	0.32/0.41	0.017	0.7 (0.56-0.87)	0.31/0.37	0.025	0.76 (0.6-0.97)	1x10^−3^
rs3885634	G	0.33/0.41	0.020	0.71 (0.57-0.89)	0.31/0.37	0.021	0.76 (0.6-0.96)	1.11x10^−3^
rs11006830	G	0.33/0.41	0.022	0.72 (0.58-0.89)	0.31/0.37	0.035	0.78 (0.61-0.98)	1.88x10^−3^

Note: P_logistic_ refers to the p-value calculated by logistic regression analysis and P of validation phase refers to chi-square p-value. P_meta_ refers to the meta-analysis of P_logistic_ of discovery phase (see Additional file 2) and P of the validation phase

We further analysed the haplotypes of 6 tag SNPs out of 14 MPP7 SNPs (see Additional file 7) and the haplotype with ‘A’ allele at the fifth position (GAAG**A**C) for rs10763643, was found to be associated as a risk haplotype for POAG (p = 6.97X10^−5^). Two different haplotypes (AAGA**G**A and AGGA**G**A) with the other allele (‘G’ for rs10763643) are associated as a protective haplotype (p = 6x10^−4^ and 3.1x10^−3^ respectively). The details are furnished in Additional file 8.

### MPP7 is downregulated in human trabecular meshwork cells upon cyclic mechanical stress

**Fig. 3:**
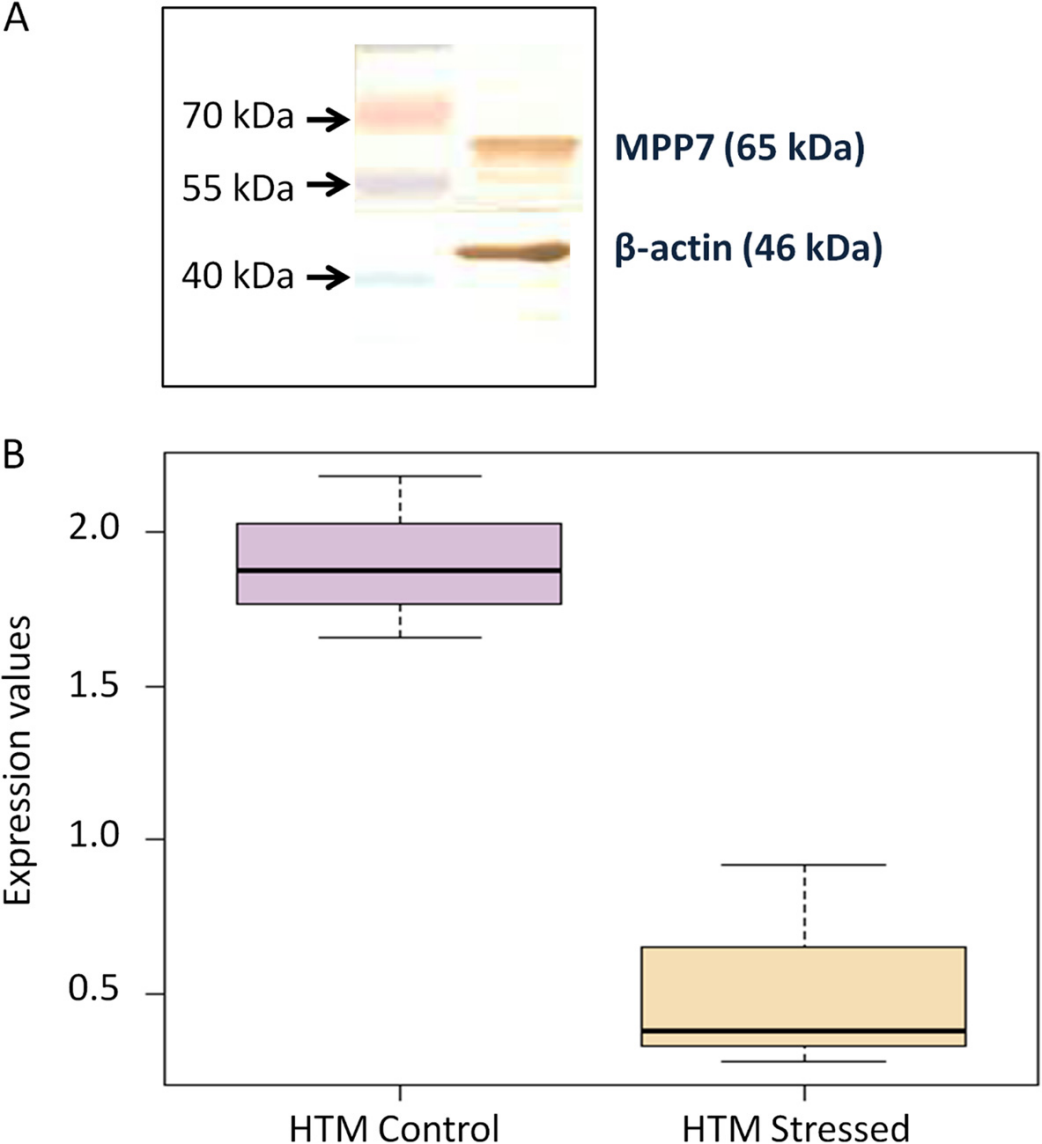
Protein expression of MPP7 in normal HTM cells and down-regulation of mRNA under cyclic mechanical stress. **a** The immunoblot shows the expression of MPP7 in HTM cell line. Thirty microgram protein was loaded on gel. β-Actin was used as loading control. Three independent experiments were done to confirm the expression. **b** This plot represents the expression of MPP7 gene transcripts in primary HTM cells with (HTM_stressed) and without cyclic mechanical stress (HTM_control) in a publicly available dataset (GSE14768). X-axis represents MPP7 shows down-regulation under cyclic mechanical stress with significant FDR-corrected p-value of 0.018 and fold change in stressed HTM was observed to be 2.6 (log Fc = −1.38)

We observed abundant expression of MPP7 protein in the HTM cells by western blot (Fig. 3a). The glaucoma phenotype, especially those associated with elevated intra-ocular pressure, is usually linked with restricted outflow of the aqueous humour through the TM - thus mimicked in vitro by cyclic mechanical stress on TM cells. A publicly available gene expression dataset at gene expression omnibus (GSE14768) of primary TM cells from donor eye, without any history of glaucoma, revealed MPP7 expression to be significantly down-regulated (FDR adjusted p-value = 0.018, fold change = 2.6) under cyclic mechanical stress as compared to the TM cells without stress (Fig. 3b).

### MPP7 is highly expressed in the ciliary processes of murine eye

**Fig. 4:**
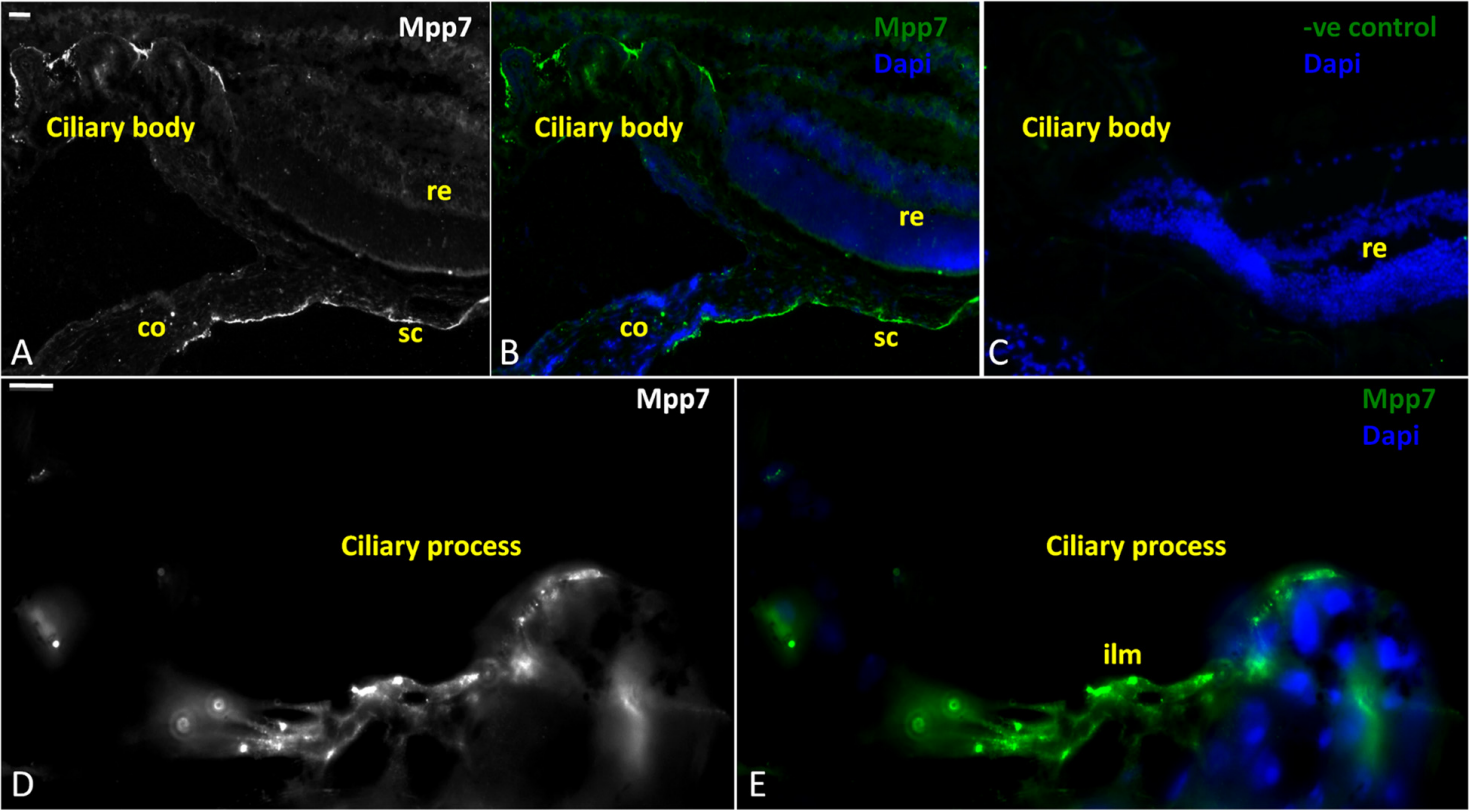
MPP7 protein expression in mouse eye by Immunofluorescence. Immunofluorescence assay showing MPP7 expression in P15 mouse eye. **a**, **b** MPP7 is highly expressed in the ciliary body as well as in the sclera. **c**: Negative control in which only secondary antibody was used. **d**,**e** Micrographs at high magnification displaying MPP7 localization in a ciliary process. Nuclei were stained using DAPI (blue). re, retina; co, cornea; sc, sclera; ilm, internal limiting membrane. Scale bar 10 μm

MPP7 protein expression was also analyzed in the murine eye by immunofluorescence. Experiments were performed on C57/BL6 mouse at P15 (15 days after birth). High expression was observed in the sclera and ciliary body (Fig. 4a, b). Within the ciliary processes the protein was mainly detected in the internal limiting membrane (Fig. 4d, e).

### Association of reported susceptibility loci of POAG in our study cohort

We checked for the association of loci that are already reported to be associated with POAG through GWA studies. Independently, the CDKN2B-AS1 locus was tested and found to be not associated in this population [11]. For all other 10 loci, we found suggestive association of multiple SNPs (see Additional file 9 and 10). Among these, one SNP of AFAP1 is associated in both discovery and validation phase (see Additional file 11). Among the reported SNPs from these loci, we found nominal association of rs4236601 in *CAV1/*CAV2 and rs7081455 of *PLXDC2* (see Additional file 9).

## Discussion

Using an unbiased two stage genome-wide screen, we suggest that *MPP7* as a potential novel candidate locus for POAG. MPP7 (membrane protein palmitoylated 7) is a member of Membrane-Associated Guanylate Kinase (MAGUK) subfamily of proteins and facilitates epithelial tight junctions formation together with Discs, Large Homolog 1 (DLG1), another MAGUK subfamily member [19, 20]. In eye, tight junctions in the non-pigmented ciliary body epithelium are crucial in barrier function responsible for ultra-filtration of plasma leading to the production of aqueous humour [21]. Rate of aqueous humour production, its content and outflow through the Trabecular Meshwork (TM) is reported to be disturbed in glaucoma [22–25]. This has also been one of the main line of disease management strategy [26] both for high and low tension glaucoma groups. The association and allele frequencies for the MPP7 SNPs were consistent when we divided the patients into high tension (IOP > 23 mm Hg) and low tension (IOP < 19 mm Hg) sub-groups and compared against the controls (see Additional file 12). This is in agreement with the possible role of MPP7 influencing aqueous humour dynamics in POAG not specific to any sub-type categorized based on IOP.

It has been recently shown by in vitro experiments that MAPK pathways in TM can be activated by ciliary epithelial cells (ODM-2) implicating crosstalk between TM and ciliary epithelium [27]. This indicates that a dysfunctional crosstalk can result in dysregulated aqueous humor outflow influencing glaucoma pathogenesis. Interestingly, we found abundant expression of MPP7 protein in human trabecular meshwork cells (Fig. 3a) and the internal limiting membrane of ciliary processes of the murine eye (Fig. 4). Further, the analysis of publicly available expression dataset has revealed that upon cyclic mechanical stress (CMS), *MPP7* is significantly down-regulated in the trabecular meshwork (Fig. 3b).

In humans, the majority of aqueous humor exits the eye via the conventional outflow pathway, composed of trabecular meshwork (TM) and Schlemm’s canal (SC) [28]. IOP is a dynamic stressor that continuously alters the biomechanical environment to which the ocular parts involved in outflow are exposed. It has been reported that cyclic IOP in perfused anterior segments of human and porcine eyes resulted in a significant decrease in outflow facility and suggest that it may result from active cellular responses to the cyclic mechanical stimulus [28]. It has been also reported that upon CMS, the family of proteins involved in regulation of cell-adhesion and cytoskeletal organization are significantly down-regulated [29]. *MPP7* knock-down causes problem in tight junction formation of epithelial cells [19] and our observation of it being down-regulated upon CMS might be an indication of a similar dysfunction of cell-cell interactions. It has been reported that other MAGUK sub-family members (e.g. ZO-1), that regulates tight junction formation are also down-regulated under elevated hydrostatic pressure on HTM cells [30]. MPP7 is known to bind MPP5, a component of crumbs complex [19]. Crumbs complex functions in epithelial cell polarity and has been shown to be involved in retinal degeneration [31]. Whether MPP7 is a crucial member for maintaining tight junctions and cell polarity of ciliary epithelium will be revealed by further functional studies.

Genetic variants in *MPP7* have also been implicated in other diseases. Reports suggest that *MPP7* is a susceptibility gene for site-specific bone mineral density and osteoporosis [32, 33]. *Okamoto N* et al. identified micro deletions at 10p11.23-p12.1 overlapping with this gene in children with unknown congenital craniofacial anomalies [34]. *MPP7* has also been implicated in intellectual disability and/or multiple congenital anomalies (ID/MCA) through identification of single gene de novo copy number variations [35]. MAGUKs have been implicated in synaptic development and plasticity including processes in the retina [36, 37]. This study suggests a novel association of MPP7 with POAG. To confirm these results, further studies need to be undertaken in larger cohorts from different populations.

## Conclusions

Based on the data of genetic association, expression in ciliary processes and downregulation in trabecular meshwork upon cyclic mechanical stress, we suggest *MPP7*, as a novel candidate gene for POAG possibly influencing the aqueous humour dynamics.

## Additional files

Additional file 1: Contains figure representing MDS plot of principal component analysis in discovery cohort samples. The arrows represent three outlier cases which were removed from final analysis. C1, C2, C3 and C4 represent four components of Principal component analysis. (TIF 1422 kb) Additional file 2: Contains a table representing the association of 494 validation phase SNPs in discovery, validation phase and meta-analysis of both. (XLSX 59 kb) Additional file 3: Contains figure representing genotype and allele error rate of individual and combined dataset of CEU and GIH populations of HAPMAP phase3. Error rates of chromosome 6 and chromosome 21 are shown as example here. The similar distribution of error rates was observed for every autosome. (TIF 646 kb) Additional file 4: Contains table representing SNPs removed from the analysis for significantly different allele frequencies in discovery and validation cohorts. (XLSX 8 kb) Additional file 5: Contains figure representing quality control of the validation data. (A) Genotype concordance of technical replicates. X-axis represents the replicate sample Id and Y-axis shows percentage genotype concordance. (B) Cluster of rs10763643 in validation cohort. The gentrain score is 0.70. (TIF 158 kb) Additional file 6: Contains figure representing manhattan plot of discovery cohort. The plot was generated from the logistic regression p-values (adjusted for population stratification) of 5,21,873 autosomal SNPs for 347 cases and 354 controls in discovery cohort. p-value of 10^−3^, taken as threshold for selection of SNPs for validation. The topmost signal is rs1294092 which was not validated in the independent validation stage. (TIF 175 kb) Additional file 7: Represents LD structure of MPP7 locus. The squares represent the tag SNPs which are also taken for haplotype association as shown in additional table 8. The tag SNPs are represented as orange squares. (TIF 4673 kb) Additional file 8: Contains table representing haplotype association of tag SNPs of MPP7 locus in East Indian population. (XLS 21 kb) Additional file 9: Contains table representing association of published SNPs form reported loci. (XLSX 11 kb) Additional file 10: Contains table representing association data of all SNPs showing suggestive association (p < 0.05) of reported loci associated with POAG. (XLS 40 kb) Additional file 11: Represents association data of *AFAP1* SNP in discovery and validation phase. (XLSX 9 kb) Additional file 12: Contains table representing association of 14 MPP7 SNPs in High tension glaucoma (HTG) and low-tension glaucoma (non-HTG) patients. (XLSX 12 kb)

## Abbreviations

POAG: primary open angle glaucoma
IOP: intra-ocular pressure
RGC: retinal ganglion cells
GWA: genome-wide association
MPP7: membrane protein palmitoylated 7
GEO: gene expression omnibus
HTM: human trabecular meshwork
CCT: Central Corneal Thickness
LD: linkage disequilibrium
CMS: cyclic mechanical stress
