# Supplementary figures and images for: Genetic association and stress mediated down-regulation in trabecular meshwork implicates MPP7 as a novel candidate gene in primary open angle glaucoma
Source: BMC Med Genomics. 2016 Mar 22;9:15. doi: 10.1186/s12920-016-0177-6 (PMC4802647; doi:10.1186/s12920-016-0177-6)

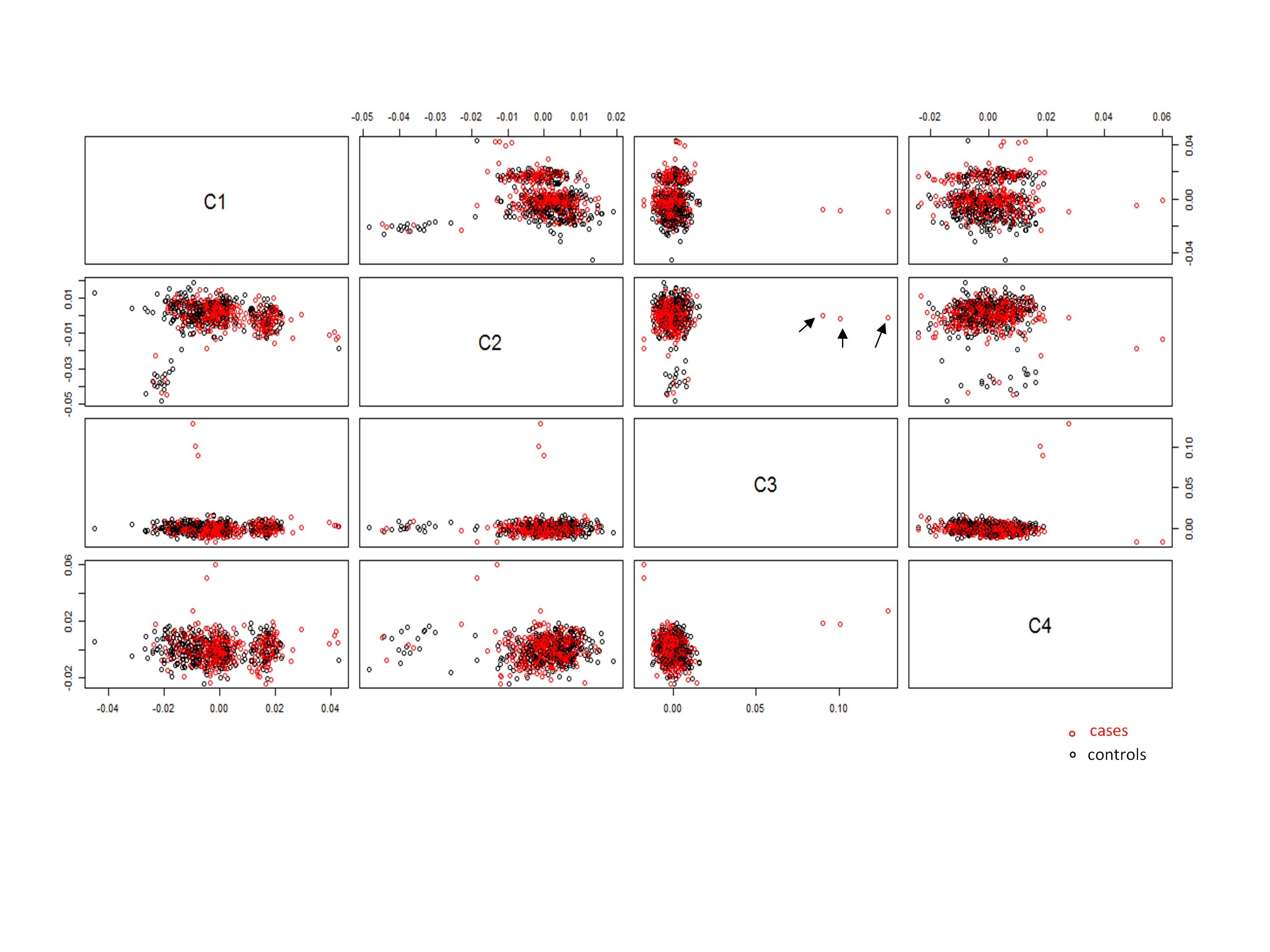

Supplement: Additional file 1: — Contains figure representing MDS plot of principal component analysis in discovery cohort samples. The arrows represent three outlier cases which were removed from final analysis. C1, C2, C3 and C4 represent four components of Principal component analysis. (TIF 1422 kb) [file 12920_2016_177_MOESM1_ESM.tif]

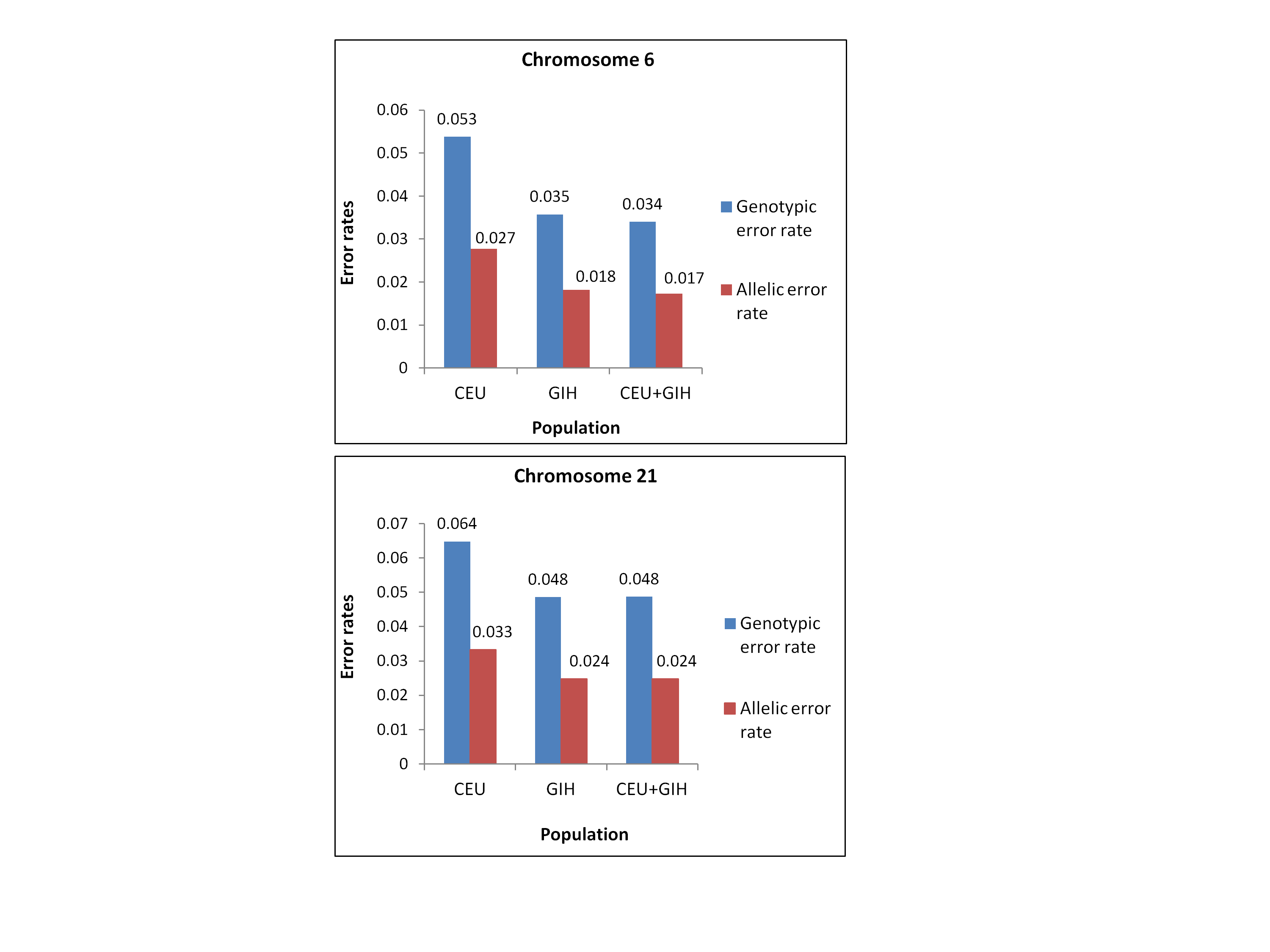

Supplement: Additional file 3: — Contains figure representing genotype and allele error rate of individual and combined dataset of CEU and GIH populations of HAPMAP phase3. Error rates of chromosome 6 and chromosome 21 are shown as example here. The similar distribution of error rates was observed for every autosome. (TIF 646 kb) [file 12920_2016_177_MOESM3_ESM.tif]

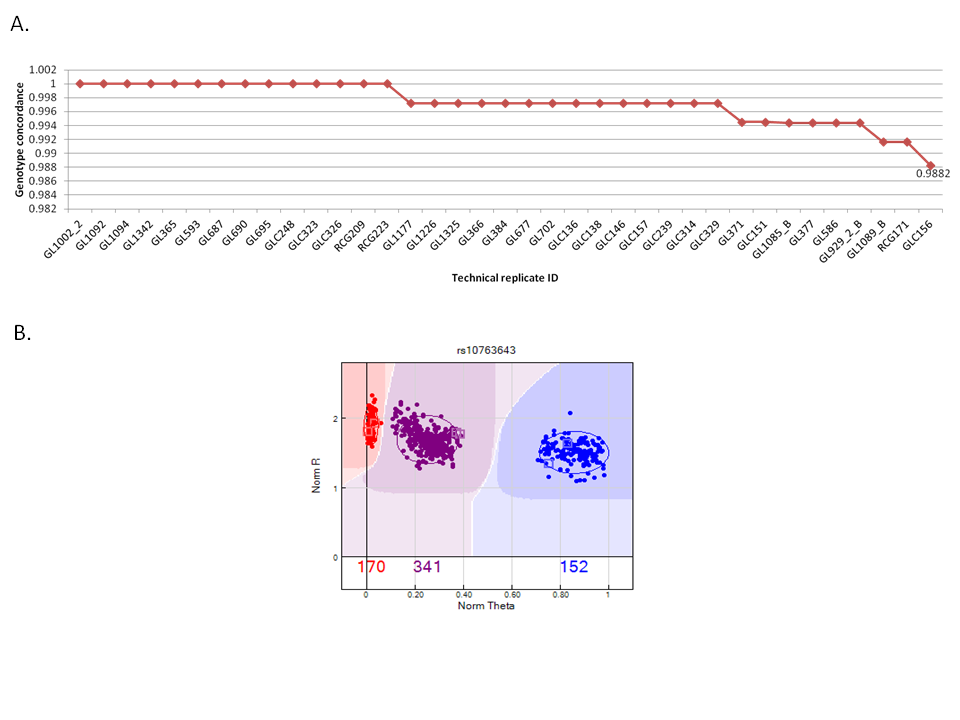

Supplement: Additional file 5: — Contains figure representing quality control of the validation data. (A) Genotype concordance of technical replicates. X-axis represents the replicate sample Id and Y-axis shows percentage genotype concordance. (B) Cluster of rs10763643 in validation cohort. The gentrain score is 0.70. (TIF 158 kb) [file 12920_2016_177_MOESM5_ESM.tif]

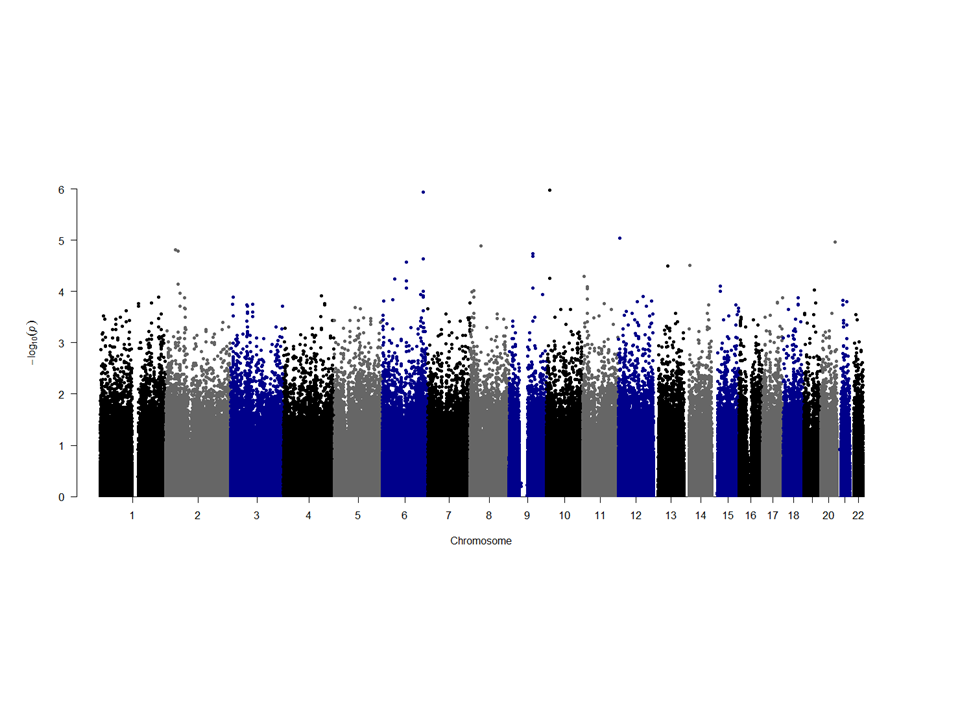

Supplement: Additional file 6: — Contains figure representing manhattan plot of discovery cohort. The plot was generated from the logistic regression p-values (adjusted for population stratification) of 5,21,873 autosomal SNPs for 347 cases and 354 controls in discovery cohort. p-value of 10−3, taken as threshold for selection of SNPs for validation. The topmost signal is rs1294092 which was not validated in the independent validation stage. (TIF 175 kb) [file 12920_2016_177_MOESM6_ESM.tif]

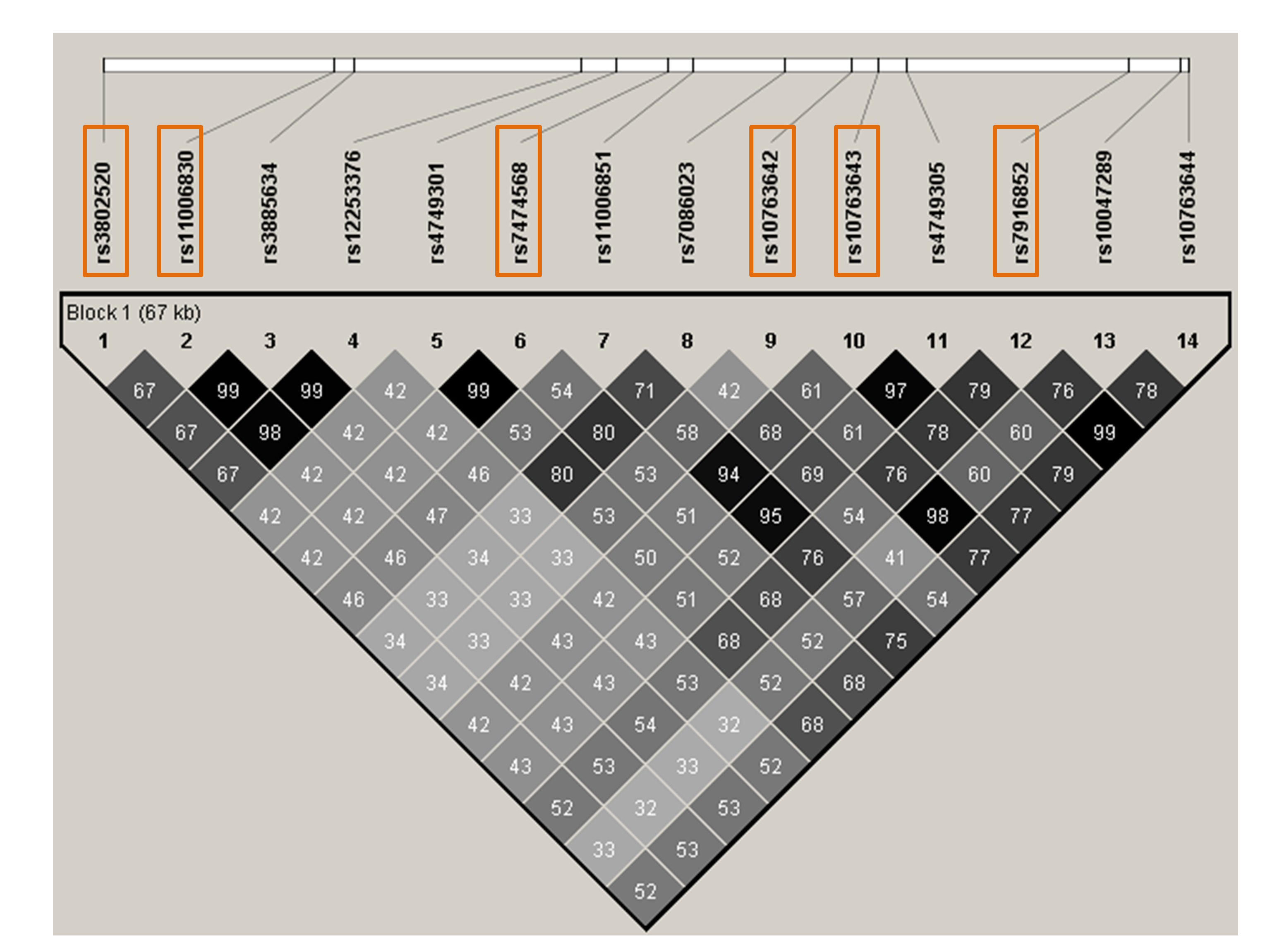

Supplement: Additional file 7: — Represents LD structure of MPP7 locus. The squares represent the tag SNPs which are also taken for haplotype association as shown in additional table 8. The tag SNPs are represented as orange squares. (TIF 4673 kb) [file 12920_2016_177_MOESM7_ESM.tif]
